# Fisetin Attenuates Doxorubicin-Induced Cardiomyopathy *In Vivo* and *In Vitro* by Inhibiting Ferroptosis Through SIRT1/Nrf2 Signaling Pathway Activation

**DOI:** 10.3389/fphar.2021.808480

**Published:** 2022-02-22

**Authors:** Danlei Li, Xiaoman Liu, Wenhu Pi, Yang Zhang, Lei Yu, Cheng Xu, Zhenzhu Sun, Jianjun Jiang

**Affiliations:** ^1^ Department of Cardiology, Taizhou Hospital of Zhejiang Province Affiliated to Wenzhou Medical University, Linhai, China; ^2^ Key Laboratory of Radiation Oncology of Taizhou, Department of Radiation Oncology, Radiation Oncology Institute of Enze Medical Health Academy, Affiliated Taizhou Hospital of Wenzhou Medical University, Linhai, China

**Keywords:** fisetin, DOX, cardiomyopathy, ferroptosis, Nrf2, SIRT1

## Abstract

Doxorubicin (DOX) is an anthracycline antibiotic that is used extensively for the management of carcinoma; however, its clinical application is limited due to its serious cardiotoxic side effects. Ferroptosis represents iron-dependent and reactive oxygen species (ROS)-related cell death and has been proven to contribute to the progression of DOX-induced cardiomyopathy. Fisetin is a natural flavonoid that is abundantly present in fruits and vegetables. It has been reported to exert cardioprotective effects against DOX-induced cardiotoxicity in experimental rats. However, the underlying mechanisms remain unknown. The present study investigated the cardioprotective role of fisetin and the underlying molecular mechanism through experiments in the DOX-induced cardiomyopathy rat and H9c2 cell models. The results revealed that fisetin treatment could markedly abate DOX-induced cardiotoxicity by alleviating cardiac dysfunction, ameliorating myocardial fibrosis, mitigating cardiac hypertrophy in rats, and attenuating ferroptosis of cardiomyocytes by reversing the decline in the GPX4 level. Mechanistically, fisetin exerted its antioxidant effect by reducing the MDA and lipid ROS levels and increasing the glutathione (GSH) level. Moreover, fisetin exerted its protective effect by increasing the SIRT1 expression and the Nrf2 mRNA and protein levels and its nuclear translocation, which resulted in the activation of its downstream genes such as *HO-1* and *FTH1*. Selective inhibition of SIRT1 attenuated the protective effects of fisetin in the H9c2 cells, which in turn decreased the GSH and GPX4 levels, as well as *Nrf2*, *HO-1*, and *FTH1* expressions. In conclusion, fisetin exerts its therapeutic effects against DOX-induced cardiomyopathy by inhibiting ferroptosis *via* SIRT1/Nrf2 signaling pathway activation.

## Introduction

Doxorubicin (DOX), a highly effective antitumor anthracycline antibiotic derived from *Streptomyces*, is commonly used for the treatment of many types of malignancies, including solid tumors, breast cancer, soft tissue sarcomas, lymphomas, and leukemia (Carvalho et al., 2009). However, the cumulative cardiotoxic effects of DOX such as those that cause irreversible degenerative cardiomyopathy and congestive heart failure limit its clinical applications (Ewer and Ewer 2015; Nebigil and Désaubry 2018). The underlying mechanisms of DOX-induced cardiotoxicity are thought to be complex and multifactorial that involve increased lipid peroxidation, oxidative stress, DNA/RNA damage, autophagy and apoptosis inhibition, and calcium homeostasis disturbance (Octavia et al., 2012; Li et al., 2016; Abdel-Daim et al., 2017). In particular, growing evidence suggests that the overproduction of reactive oxygen species (ROS) and increased oxidant-induced mitochondrial damage are crucial for the development of DOX-induced cardiotoxic effects (Christiansen 2011; Carvalho et al., 2014). Additionally, ROS and mitochondrial dysfunction are the key factors for ferroptosis, which is a newly discovered type of regulated cell death characterized by the accumulation of a large amount of iron and lipid peroxidation in cells (Dixon et al., 2012). Glutathione peroxidase 4 (GPX4) is considered to be a key marker of ferroptosis because it directly reduces toxic lipid peroxides to nontoxic lipid alcohols (Forman et al., 2009; Ingold et al., 2018; Forcina and Dixon 2019). Ferroptosis has been reported to be associated with various diseases, including glutamate-induced neuronal cell death and neurodegenerative diseases (Skouta et al., 2014; Yan and Zhang 2019), drug-induced liver injury (Lorincz et al., 2015), acute kidney injury (Friedmann Angeli et al., 2014; Wang Y et al., 2021), and myocardial injury induced by ischemia/reperfusion (I/R) and antitumor drugs such as DOX (Yang et al., 2014; Fang et al., 2019; Tadokoro et al., 2020). Researchers have also discovered that natural flavonoids could be used to ameliorate the DOX-induced cardiotoxicity based on flavonoids’ antioxidant, anti-inflammatory, and anti-apoptosis potentials (Guo et al., 2020; Navarro-Hortal et al., 2020; Qi et al., 2020; Sun et al., 2020); however, supporting evidence for the involvement of flavonoids in DOX-induced ferroptotic myocardial cell death is lacking.

Fisetin (3,3′,4′,7-tetrahydroxyflavone) is one of the flavonoids that is present abundantly in grape seed, apple, strawberry, onion, cucumber, and persimmon. Because of the similarity of its molecular structure with the benzoquinone pharmacophore, fisetin exhibits numerous biological activities including antioxidant, anti-mitosis, anti-inflammation, and anticancer activities (Zheng et al., 2008; Currais et al., 2014) and protects against the oxidative stress-related degenerative diseases such as diabetes and cardiovascular diseases (Manach et al., 2005; Scalbert et al., 2005). Mechanistically, several studies have reported that fisetin can activate the SIRT1 (Sirtuin 1) signaling pathway in experimental models such as the osteoarthritis model (Zheng et al., 2017), tunicamycin-mediated cell death in PC12 cells (Yen et al., 2017), lead-induced neurotoxicity (Yang et al., 2019), and aging-induced neurodegeneration (Singh et al., 2018). Fisetin also regulates the Nrf2 (nuclear factor erythroid 2-related factor 2) expression (Kim et al., 2016; Zheng et al., 2017; Zhang et al., 2019). Nrf2 has been reported to be associated with ferroptosis in lung cancer (Liu et al., 2020), LPS-induced acute lung injury (Li et al., 2021), and neurodegenerative diseases (Song and Long 2020). A recent study revealed that ulinastatin exerted a hepatoprotective effect against ferroptosis by activating the SIRT1/Nrf2 signaling pathway (Wang C et al., 2021). However, whether fisetin protects the cardiomyocytes against DOX-induced ferroptosis through a SIRT1-dependent mechanism is not yet known.

Nrf2 is considered a master regulator of the antioxidant response as many of its downstream target genes and enzymes are responsible for correcting or preventing intracellular redox imbalances (Kerins and Ooi 2018). The antioxidant defense genes such as heme oxygenese-1 (*HO-1*), ferritin heavy chain 1 (*FTH1*), ferritin light chain (*FTL*), ferroportin (*FPN*), and *NADPH* are considered as the Nrf2 downstream target genes and play crucial roles in the development of ferroptosis (Anandhan et al., 2020; Dong et al., 2020; Song and Long 2020). Nrf2 can be activated by SIRT1 deacetylation (Fan et al., 2017). SIRT1, a well-known stress response protein, plays key roles in different cellular and physiological functions such as mitochondrial biogenesis, cell injury, and cell death including ferroptosis. SIRT1 has been shown to increase cell resistance to DOX-induced cardiotoxicity (Yuan et al., 2018; Sun et al., 2020). Furthermore, numerous studies have indicated that SIRT1 could manifest its antioxidative effects *via* the activation of Nrf2 in various diseases such as diabetes and rheumatoid arthritis (Zhang X et al., 2018; Samimi et al., 2019; Wang et al., 2020). However, none of the studies have investigated the role of SIRT1/Nrf2 in DOX-induced ferroptosis in cardiomyopathy.

Based on these promising findings, we postulated that fisetin protects against doxorubicin-induced cardiotoxicity and investigated the molecular mechanism of fisetin in ferroptosis inhibition *in vivo* and *in vitro* in the present study.

## Materials and Methods

### Animals and Treatment

All animal care and experimental procedures were approved by the Animal Care and Use Committee of Taizhou Hospital of Zhejiang University (Taizhou, China). Eight-week-old Waster rats (24 males, body weight 190–220 g) were obtained from the Experimental Animal Center of Basi Medicine, Zhejiang Chinese Medical University. The rats were kept in a room with a circumambient temperature of 23 ± 1°C, a 12-h dark/light cycle, and a relative humidity of 55% ± 5%. The rats were acclimatized to the laboratory for 1 week before the studies.

All experimental rats were randomly divided into four groups (6 rats/group): (I) Control group, (II) DOX group, (III) DOX + fisetin1 (fisetin 20 mg/kg/day) group, and (IV) DOX + fisetin2 (fisetin 40 mg/kg/day) group. Group (I) received saline solution (0.9% NaCl) by gavage for 6 weeks (2 ml/day). The groups (II), (III), and (IV) were administered 2.5 mg/kg DOX (Cat# HY-15142A, MedChemExpress, USA) through injection *via* the tail vein once a week (at day 7 of the week) for 6 weeks (total dose: 15 mg/kg body weight) to induce cardiotoxicity. The groups (III) and (IV) were respectively administered fisetin (Cat.HY-N0182, MedChemExpress, USA) 20 mg/kg/day and 40 mg/kg/day (dissolved in 0.1% dimethyl sulfoxide), intragastrically once daily. The doses of fisetin and DOX were determined based on previously described protocols (Yang et al., 2019; Sun et al., 2020).

Tail vein injection: The rat was taken from the housing cage and kept in a 32–35°C chamber for 10–15 min; then, the rat was introduced onto the tail vein injection device. The injection site of the tail was wiped with a 70% alcohol pad. The needle of an insulin syringe was inserted into the skin in a 10–15° angle about 2–4 mm to penetrate the tail vein, and 500 μg DOX solution was slowly injected. The needle was removed quickly, and the injection site was pressed firmly to prevent the backflow of the injected DOX solution and/or blood. Then, the rat was returned to its cage and monitored for at least 5 min to ensure the rat has no further bleeding.

### Echocardiography

At the end of treatment, the rats were anesthetized using isoflurane (RWD, Shenzhen, China), and two-dimensional M-mode echocardiography was performed using SONOS 5500 ultrasound (Philips Electronics, Amsterdam, The Netherlands) equipped with a 15-MHz linear array ultrasound transducer. The left ventricular ejection fraction (LVEF), left ventricular fraction shortening (LVFS), left ventricular end-diastolic internal dimension (query) (LVIDd), and left ventricular end-systolic internal dimension (query) (LVIDs) were measured in five consecutive cardiac cycles, and the values were averaged to obtain a representative result for each parameter.

### Heart Tissue Histological Analysis

The animals were sacrificed using isoflurane overdose. Heart tissues were cleaned with phosphate-buffered saline (PBS) and fixed with 4% paraformaldehyde. The fixed tissue was embedded in paraffin and cut into 5-µm thick sections for the histological analysis. The tissue sections were stained with hematoxylin and eosin (H&E) (G1120, Sbjbio Life Sciences, China), Masson’s trichrome (G1340-7, Sbjbio Life Sciences, China), and wheat germ agglutinin (WGA) (Sigma-Aldrich, USA), according to the kit standard protocols. The stained sections were observed and imaged under a Nikon Eclipse Ti2 fluorescence microscope; the positively stained areas were analyzed by ImageJ software (NIH Image, Bethesda, MD, USA) to evaluate the cardiac morphology and the extent of myocardial fibrosis and heart hypertrophy.

### Immunohistochemical Analysis

The cardiac sections were deparaffinized and dehydrated using xylene and different concentrations of alcohol; the slides were rinsed with distilled water. The deparaffinized sections were boiled for 20 min in the antigen-repair buffer (0.01 M citric acid solution pH 6.0) for facilitating antigen repair. Then, the slides were cooled down to room temperature and treated with 3% H_2_O_2_ solution for 30 min to block the endogenous peroxidase activity. Subsequently, the slides were placed in PBS with 1% BSA for 30 min. The slides were incubated overnight at 4°C with the primary antibodies against SIRT1 (mouse, 1:200, ab110304, Abcam) and Nrf2 (mouse, 1:250, ab89443, Abcam). To determine the presence of proteins, the sections were stained using the 3,30-diaminobenzidine (DAB) (Absin, Shanghai, China) staining solution. The stained sections were photographed at 200× magnification by using an Olympus CKX41 microscope, and the positive signals were indicated by the brownish yellow color.

### Cell Culture and Drug Treatment

The H9c2 cell line was obtained from the Cell Bank of Chinese Academy of Sciences (Shanghai, China). The cells were cultured in Dulbecco’s Modified Eagle Medium (DMEM; Sigma, USA) supplemented with 10% fetal bovine serum (FBS; Gibco, USA) and 1% penicillin/streptomycin at 37°C in a humidified incubator with 5% CO_2_. The H9c2 cells were equally seeded into 6-well culture plates and assigned to the following four groups: (I) control, (II) fisetin, (III) DOX, and (IV) DOX + fisetin. Each group was tested in duplicate. When the seeded cells reached up to 70–80% confluence, they were treated with fisetin (40 μm), DOX (1 μm), and DOX + fisetin for 24 h, whereas the control cells were treated with DMSO. The SIRT1-knockdown H9c2 cells also performed the same.

### siRNA Transfection

SIRT1 and Nrf2 siRNAs (small interfering RNAs) were purchased from GenePharma, (Shanghai, China), and the SIRT1 siRNA sequences are as follows: sense primer 5-GCAGAUU AGUAAGCGUCUUTT-3′ and antisense primer 5′-AAG​ACG​CUU​ACU​AAU​CUG​C TT-3′. The Nrf2 siRNA sequences were the following: sense primer 5′-GUAAGAAGCCAG AUGUUAA-3′ and antisense primer 5′-UUC​UCC​GAA​CGU​GUC​ACG​UTT-3′. Both SIRT1 and Nrf2 siRNAs and control siRNA (100 pm) were transfected into the H9c2 cells by using Lipofectamine 3,000 transfection reagents (Cat# L3000-008, Invitrogen, USA), according to the manufacturer’s protocol. The SIRT1 and Nrf2 expression level was determined after 48 h of transfection.

### Cell Viability Assay

The H9c2 cells were seeded into 96-well plates at a density of 1 × 10^4^ cells/well. After different drug intervention treatments for 24 h, the cells were treated with the Cell Counting Kit-8 (CCK-8) solution (MedChem Express, USA) for 1 h at 37°C. The absorbance was measured at 450 nm with a microplate reader (Thermo Scientific™ Multiskan™ FC). Relative cell viability was calculated as the ratio of the absorbance of each treatment group to that of the control group.

### MDA Detection Assays

The MDA contents in cell and heart tissue lysates were measured using a lipid peroxidation (MDA) assay kit (Cat#A003-1, Nanjing Jiancheng Bioengineering Institute, China). Briefly, this method is based on the reaction of MDA in the cells and tissues with thiobarbituric acid (TBA) and generates an MDA-TBA adduct, which can be quantified colorimetrically using a microplate reader (OD = 532 nm). Cells or heart tissues after treatment of various agents (method 2.1 and 2.5) were homogenized and sonicated in lysis buffer. The buffer volume is 100 μl/1*10^6^ cells or 100 μl/10 mg tissues according to the manufacturer’s protocol. The lysate was centrifuged at 13,000 rpm/min for 15 min; then, the supernatant was transferred to a new 1.5 ml centrifuge tube. The protein concentration in the sample was analyzed by using a bicinchoninic acid protein assay kit (BioSource, Camarillo, CA, USA). A total of 100 μL supernatant was mixed with 200 μl TBA solution in a testing tube, alongside an MDA standard solution (10 nmol/ml), and distilled water (standard blank) was mixed with the TBA solution in another blank tube. Each mixture was incubated at 95°C for 1 h and cooled down to room temperature (RT) in an ice bath. After a centrifugation of 3,000 r/min for 15 min, a total of 200 μl supernatant was moved into a 96-well microplate, and the MDA-TBA adduct concentration was measured at OD = 532 nm. The MDA content (nanomole per microgram protein) was calculated according to the following formula: (the absorbance of the sample-the absorbance of standard blank/the absorbance of the standard sample-the absorbance of standard blank) x standard concentration (10 nmol/ml) x sample dilution factor/protein level of the sample (mgprot/ml) (Liu et al., 2010; Mao et al., 2012; Zhu et al., 2014).

### GSH Detection Assay

The relative GSH concentration in cells and heart tissues were assessed by using a GSH Detection Assay Kit (Cat#A006-2-1, Nanjing Jiancheng Bioengineering Institute, China). The kit is based on an enzymatic method in the presence of GSH which reacts with 5,5′-dithiobis-(2-nitrobenzoic acid) (DTNB) to form a 2-nitro-5-thiobenzoic acid (TNB) chromophore. The reduction of the chromophore releases a strong yellow signal which can be quantified spectrophotometriclly by using a microplate reader (OD = 405 nm) (Zhang et al., 2009; Caito and Aschner 2015). Cells and tissues were similarly prepared, as mentioned previously (MDA assay). A total of 100 μL sample supernatant, a GSH standard solution (20 umol/L), and distilled water (standard blank) were mixed with 100 μg reaction buffer which contained DTNB, according to the manufacturer’s protocol. The mixture was kept on RT for 5min; then, the total mixture was used for the measurement of GSH concentration at OD = 405 nm. The GSH content (micromole per gram protein) was calculated by the same formula, as mentioned previously.

### ROS Detection Assay

The cellular reactive oxygen species (ROS) assay was performed following the manufacturer’s instructions (Cat# E004-1-1, Nanjing Jiancheng Bioengineering Institute, China). The ROS assay kit is based on the diffusion of 2′,7′ –dichlorofluorescein diacetate (DCFH-DA) into the cell. It is then deacetylated by cellular esterases, and the product is later oxidized by ROS into 2′, 7′ –dichlorofluorescein (DCF), which is highly fluorescent and is easily detected using a microplate reader at 525 nm, according to the manufacturer’s protocol. The H9c2 cells were plated into 6-well culture plates at a density of 2 × 10^5^ cells/well. When the culture reached 70–80% confluency, cells were treated with different drugs for 24 h, and then, they were incubated with the ROS working reagents at 37°C for 45 min in the dark for ROS detection. To detect the ROS levels in heart tissues, cardiomyocytes were isolated from fresh heart tissues by using collagenase II digestion and followed the aforementioned method, and then, ROS levels were detected using the microplate reader at a wavelength of 525 nm.

### Iron Assay

The intracellular ferrous iron level was determined using the iron assay kit (Abcam, ab83366), according to the manufacturer’s instructions (Wang K et al., 2021).

### Cytoplasmic and Nuclear Protein Extraction

The H9c2 cells were seeded onto 6-well plates at a density of 4 × 10^5^ cells/well. After growing overnight, different drug intervention treatments were performed for 24 h; then, cells were washed with ice cold PBS and collected in 1.5 ml tubes. Cell cytoplasmic and nuclear proteins were extracted by using the NE-PER Nuclear and Cytoplasmic Extraction Reagents kit (cat# 78833, Thermo Fisher). The procedure was carried out according to the manufacturer’s protocols stepwise (Hung et al., 2007).

### Immunofluorescence Staining

The SIRT1 expression in the H9c2 cells was visualized through immunofluorescence (IF) staining. The H9c2 cells were cultured overnight on gelation-coated glass coverslips and treated with fisetin, DOX, and DOX + Fisetin for 24 h. Then, the cells were fixed in 4% paraformaldehyde for 10 min, washed thrice with PBS for 5 min/each, and permeabilized with 0.2% Triton X-100 in PBS for 15 min. The slides were first incubated with sheep serum for 30 min at room temperature to block nonspecific binding and then incubated overnight with SIRT1 (mouse, 1:1,000; ab110304, Abcam), Nrf2 (mouse, 1:100 ab89443, Abcam), and HO-1 (rabbit, 1:200, ab68477, Abcam) antibodies at 4°C. The cells were washed thrice with PBS to eliminate unbound antibodies and incubated with Alexa Fluor 488 goat anti-mouse and goat anti-rabbit IgG (Life Technologies, USA) secondary antibodies for 1 h at room temperature. Then, the cells were counterstained with 1 μg/ml DAPI for nuclear staining (D9564; Sigma-Aldrich, USA) and analyzed by fluorescence microscopy. All fluorescence images were obtained using the Nikon Eclipse Ti2 fluorescence microscope.

### Western Blotting Analysis

Total proteins were extracted from the treated cardiac H9c2 cells and heart tissues using the RIPA lysis buffer (Beyotime, China) containing the phosphatase inhibitor cocktail I (MedChemExpress, USA). The protein concentrations were determined using the BCA protein kit (Beyotime, China), and all protein samples were separated through 10% SDS-PAGE and transferred to polyvinylidene fluoride (PVDF) membranes (Millipore, MA). The blots were blocked with 5% skimmed milk for 1 h at room temperature and then incubated overnight at 4°C with the specific primary antibodies at appropriate dilutions. The primary antibodies used for the experiment were GPX4 (rabbit, 1:1,000, ab125066, Abcam), SIRT1 (mouse, 1:2,000, ab110304, Abcam), Nrf2 (mouse, 1:1,000, ab89443, Abcam), Keap1 (mouse, 1:1,000, ab119403, Abcam), HO-1 (rabbit, 1:1,000, ab68477, Abcam), FTH1 (rabbit, DF6278, Affinity Biosciences, China), FTL (rabbit, 1:2,000, ab69090, Abcam), FPN (rabbit, 1:1,000, NBP1-21502, Novus Biologicals, USA), TfR1 (mouse, 1:500, ab269513, Abcam), Lamin B (rabbit, 1:1,000, ab16048, Abcam), and GAPDH (rabbit, 1:5,000, AF1186, Beyotime). The blots were washed and incubated for 1 h at room temperature with the HRP-conjugated secondary antibodies (1:5,000, Santa Cruz, USA) and developed with enhanced chemiluminescence reagents (Cat# 34580, Thermo Fisher, USA). Densitometric quantification was performed by ImageJ analysis software (National Institutes of Health, Bethesda, MD, USA).

### Real-Time PCR Assay

Total RNA was isolated from the treated H9c2 cells by using the TRIzol reagent (Invitrogen, USA), according to the manufacturer’s instructions. cDNA was synthesized from total RNA (1 µg) by using the SuperScript First-strand RNA reverse transcription kit (Cat# 11904–018 Invitrogen USA). Real-time PCR was performed in a 7,300 plus real-time PCR system (Applied Biosystems, Thermo Fisher Scientific, USA) with the SYBR Green Master mix (Cat#A25742, Applied Biosystems, Thermo Fisher Scientific, USA) by using gene-specific primers. The reactions were allowed to run in triplicates, and *GAPDH* was used for normalization. The primers used for PCR are as follows: Nrf2 (forward primer) 5′-cag​cat​gat​gga​ctt​gga​gt-3′, Nrf2 (reverse primer) 5′-tgt​tcc​ttc​tgg​agt​tgc​tc-3′; HO-1 (forward primer) 5′-gat​ttg​tct​gag​gcc​ttg​aa-3′, HO-1 (reverse primer) 5′-aga​ctg​ggt​tct​gct​tgt​tg-3′; FTH1 (forward primer) 5′-act​gat​gaa​gct​gca​gaa​cc-3′, FTH1 (reverse primer) 5′-gtg​ggg​atc​att​ctt​gtc​ag-3′; FTL (forward primer) 5′-ctc​ctc​gag​ttt​cag​aac​ga-3′, FTL (reverse primer) 5′-gct​ttc​cag​gaa​gtc​aca​ga-3′; FPN (forward primer) 5′-tga​cct​cag​caa​aat​tcc​tc-3′, FPN (reverse primer) 5′-tct​ggg​cca​ctt​taa​gtc​tg-3′; GAPDH (forward primer) 5′-tct​ctg​ctc​ctc​cct​gtt​ct-3′, and GAPDH (reverse primer) 5′-atc​cgt​tca​cac​cga​cct​tc-3′.

### Statistical Analysis

Statistical analyses were performed by GraphPad Prism software 8.0 (San Diego, CA, USA). All results are expressed as mean ± standard deviation (SD). The differences between the groups were assessed by one-way analysis of variance (ANOVA) with Tukey’s *post hoc* analysis. A *p* value of <0.05 was considered statistically significant. All assays were repeated at least three times.

## Results

### Fisetin Exerted a Protective Effect Against DOX-Induced Cardiac Tissue Damage *In Vivo*

**FIGURE 1:**
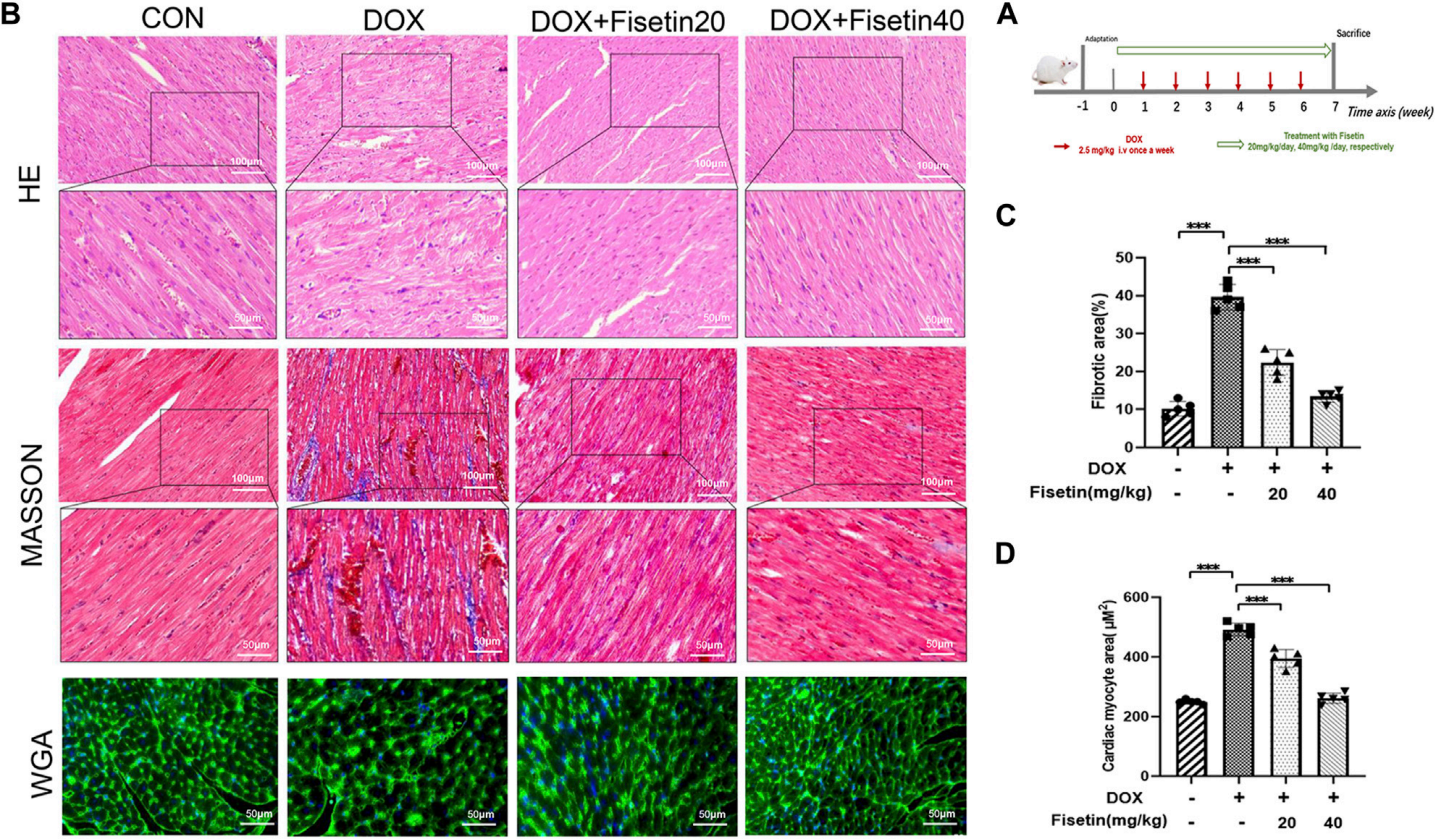
Fisetin exerts a protective effect in DOX-induced cardiac tissue damage in rats. **(A)**. Experimental design for evaluation of the protective effect of fisetin on DOX-induced cardiomyopathy in rats (*n* = 6 per group). **(B)**. Representative images of H&E staining, Masson’s staining, and WGA staining for assessment of cardiac injury, myocardial fibrosis, and cardiac hypertrophy in rats after treatment of DOX and/or fisetin (200X and 400X, Scale bar = 100 and 50 μm). **(C)**. Quantification of the fibrotic area from Masson’s staining sections and **(D)** Cardiomyocyte hypertrophy from WGA staining sections (*n* = 5). The values are presented as mean ± SD. ***p < 0.001; DOX, doxorubicin; H&E, hematoxylin and eosin; WGA, wheatgerm agglutinin.

Doxorubicin was used to induce chronic cardiac injury in the rats through tail vein injection, as reported previously (Sun et al., 2020) (Figure 1A). The protective effect of fisetin against DOX-induced cardiac tissue damage was first evaluated *in vivo*. As shown in Figure 1B, H&E staining of the rat cardiac tissues revealed that DOX caused marked histopathological damages including capillary congestion, interstitial edema, and inflammation infusion. The degree of myocardial fibrosis was evaluated in cardiac sections through Masson’s staining. Compared with the DOX-treated rats, fisetin treatment significantly decreased the histological alterations induced by the DOX administration (*p* < 0.05); the quantification result is presented in Figure 1C. DOX caused cardiac hypertrophy, which was determined through WGA staining. However, treatment with fisetin abolished this effect (Figure 1D). Moreover, the high-dose fisetin group exhibited a better protective effect than the low-dose group (Figures 1C, D).

### Fisetin Attenuated DOX-Induced Myocardial Dysfunction *In Vivo*

**FIGURE 2:**
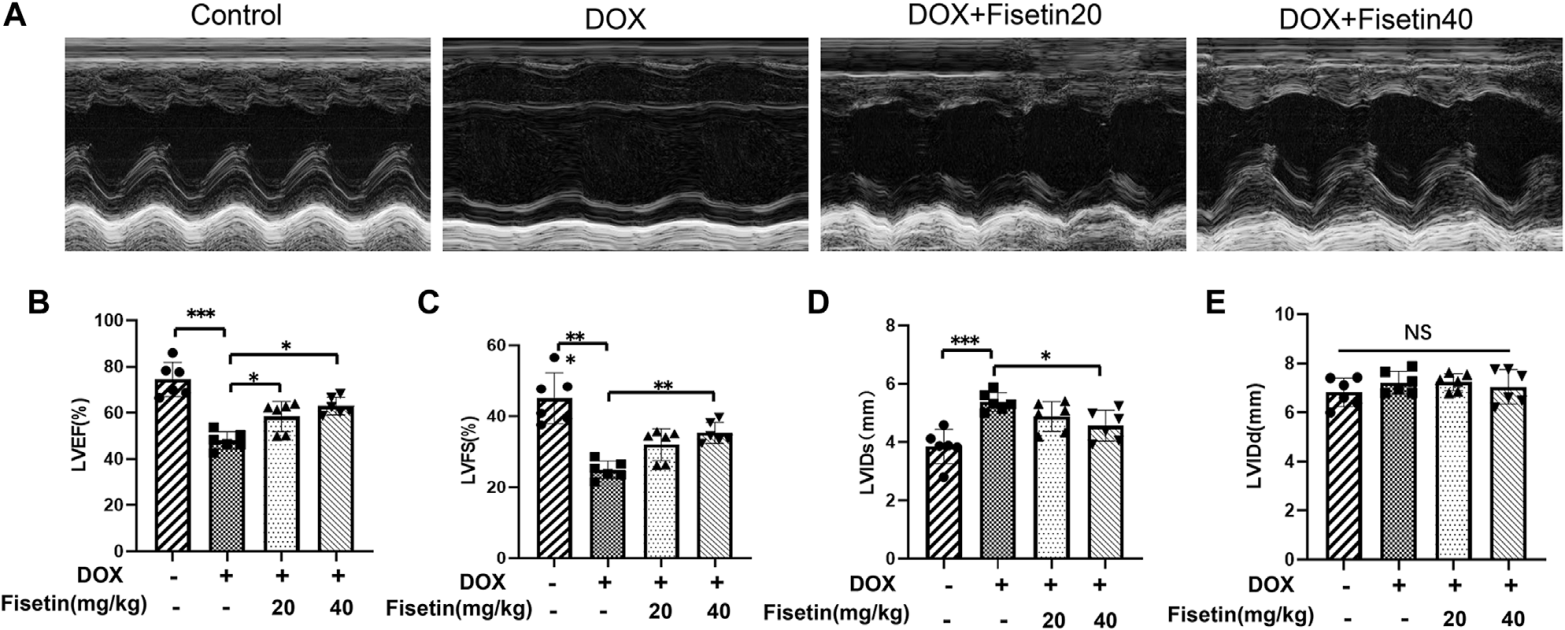
Fisetin attenuates DOX-induced myocardial dysfunction in rats. **(A)**. Representative echocardiogram images of each group. Results of **(B)** LVEF, **(C)** LVFS, **(D)** LVIDs, and **(E)** LVIDd in four group experimental rats (*n* = 6 per group). The results showed the effect of fisetin on attenuating DOX-induced LV dysfunction. The values are presented as mean ± SD. *p < 0.05; **p < 0.01; ***p < 0.001; NS, no significance; LV, left ventricular; LVEF, LV ejection fraction; LVFS, LV fractional shortening; LVIDs, LV end-systolic internal dimension; LVIDd, LV end-diastolic internal dimension.

The cardiac function of each group was evaluated through external echocardiography. As shown in Figure 2, DOX significantly impaired heart function by decreasing the LVEF and LVFS (Figures 2B,C) and by increasing the LVIDd and LVIDs (but not significantly) (Figures 2D,E), which resulted in serious left ventricle dysfunction. LVEF and LVFS were markedly increased in both DOX + fisetin groups compared with those in the DOX group (Figures 2B,C), whereas LVIDs were decreased significantly in the DOX + fisetin co-treatment group, which indicated that fisetin protected against DOX-induced cardiac (left ventricle) dysfunction *in vivo*.

### Fisetin Attenuated DOX-Induced Ferroptosis *In Vivo* and *In Vitro*

**FIGURE 3:**
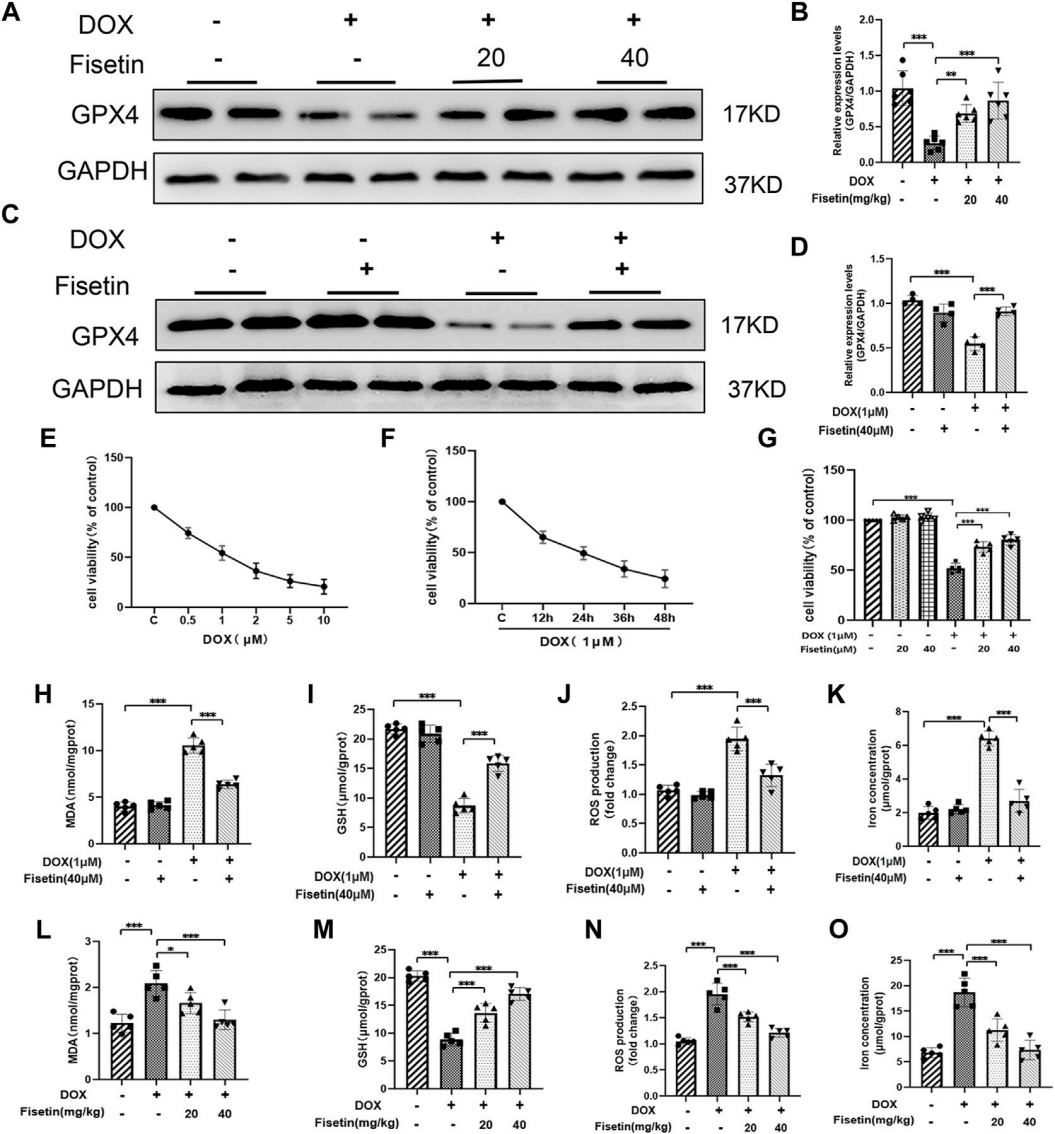
Fisetin attenuates DOX-induced ferroptosis. **(A)** Western blot results of GPX4 levels in rat heart tissues. GAPDH was used as normalization control. **(B)** Quantification of the GPX4 level from Western blot **(A)**. **(C–D)** Western blot results of GPX4 levels in H9c2 cells; the expression was quantified by ImageJ analysis. **(E)** Results of the CCK-8 assay. H9c2 cells exposed to different concentrations of DOX for 24 h. **(F)** Cell survival rate of H9c2 cells exposed to 1 µM DOX using CCK-8 assay. **(G)** Cell survival rate of co-administration of DOX + fisetin (20 and 40 µM) in H9C2 cells. **(H–K)** MDA, GSH, ROS, and iron concentration levels in cells in different groups. **(L–O)** MDA, GSH, ROS, and iron concentration levels in heart tissues. The values are presented as mean ± SD. *p < 0.05; **p < 0.01; ***p < 0.001; NS, no significance; GPX4, glutathione peroxidase 4; GSH, glutathione; ROS, reactive oxygen species; MDA, malondialdehyde; CCK-8, Cell Counting Kit-8.

To further investigate the protective role of fisetin against ferroptosis in DOX-induced cardiomyopathy, we assessed the protein expression levels in the DOX-treated rat heart tissues through Western blotting. GPX4 inhibition is one of the effective mechanisms of ferroptosis (Dixon et al., 2012). As shown in Figure 3, the GPX4 expression levels were decreased in the DOX group. Intriguingly, fisetin upregulated the expression of GPX4 *in vivo* (Figures 3A,B). Subsequently, the cytoprotective effect of fisetin was examined *in vitro* using the H9c2 rat cardiomyocyte cell model. The H9c2 cells were cultured with DOX at various concentrations (0, 0.5, 1, 2, 5, and 10 μM) for 24 or 48 h; cell viabilities of the cultured cells were significantly reduced with 1 µm DOX treatment in a dose- and time-dependent manner (Figures 3E–G). According to the CCK-8 assay result, the 1 μM concentration of DOX and the 40 μM-dose of fisetin for 24 h were selected for further experiments. To further investigate the role of ferroptosis in DOX-induced cytotoxicity, we treated the H9c2 cells with DOX (1 µM), following which the GPX4 expression was downregulated. As expected, fisetin upregulated the expression of GPX4 *in vitro* (Figures 3C,D). Because lipid peroxidation is a characteristic of ferroptosis, GSH plays a key role in maintaining the oxidation balance. We next determined the levels of the lipid-associated products MDA, ROS, and GSH *in vivo* and *in vitro*. Treatment with DOX resulted in an increase in the lipid ROS, MDA levels, and a decrease in the GSH level, whereas the treatment with fisetin blocked the effects of DOX on MDA, GSH, and lipid ROS by increasing the GSH level and decreasing the MDA, ROS levels in both rats and H9c2 cells (Figures 3H–J, L–N). In addition, we confirmed that the intracellular iron levels significantly increased, and ferroptosis occurred after DOX administration, whereas fisetin can abolish this effect (Figures 3K, O). These results suggested that DOX could induce ferroptosis and that fisetin could attenuate DOX-induced ferroptosis *in vivo* and *in vitro*. In order to confirm that ferroptosis was induced by DOX in H9c2 cells, Fer-1, a ferroptotic inhibitor, was used against DOX. The results were similar to fisetin administration, which further indicated that fisetin can ameliorate DOX-induced ferroptosis *in vitro*. (Supplementary Figure S1).

### Fisetin Inhibited DOX-Induced Ferroptosis by Regulating Nrf2 in Rats and H9c2 Cells

**FIGURE 4:**
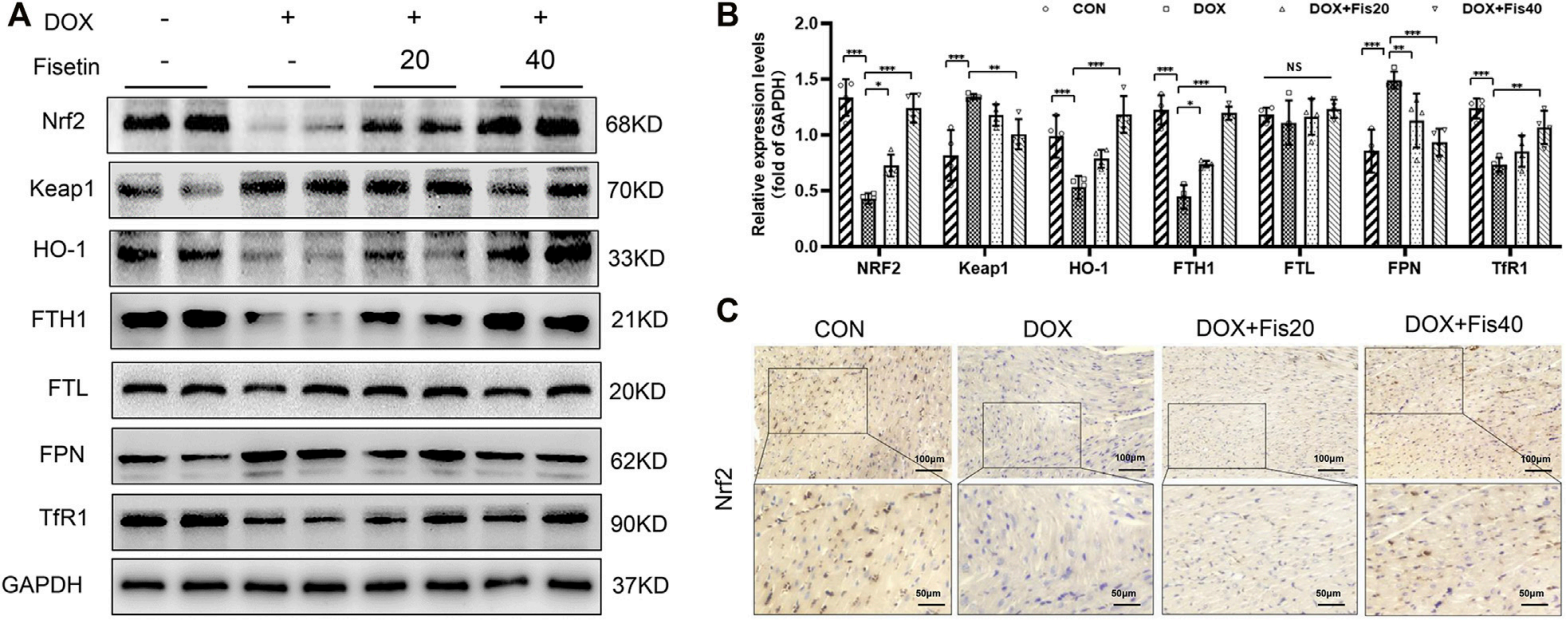
Fisetin protects against DOX-induced ferroptosis *via* regulating Nrf2 in rats. **(A)**. Western blot results of Nrf2 and Keap1, HO-1, FTH1, FTL, FPN, and TfR1 protein in control and different drug-treated rat heart tissues. **(B)**. Quantification of **(A)**. **(C)**. Expression of Nrf2 was detected by immunohistochemistry (IHC) (Representative images, 200X and 400X, Scale bar = 100 and 50 μm, *n* = 6 rats per groups) in cardiac tissue of each group. The values are presented as mean ± SD. *p < 0.05; **p < 0.01; ***p < 0.001; NS, no significance; DOX, doxorubicin. Nrf2, nuclear factor erythroid 2-related factor 2; Keap1, Kelch-like ECH-associated protein 1; HO-1, heme oxygenase-1; FTH1, ferritin heavy chain 1; FTL, ferritin light chain; FPN, ferroportin; TfR1, transferrin receptor 1.

**FIGURE 5:**
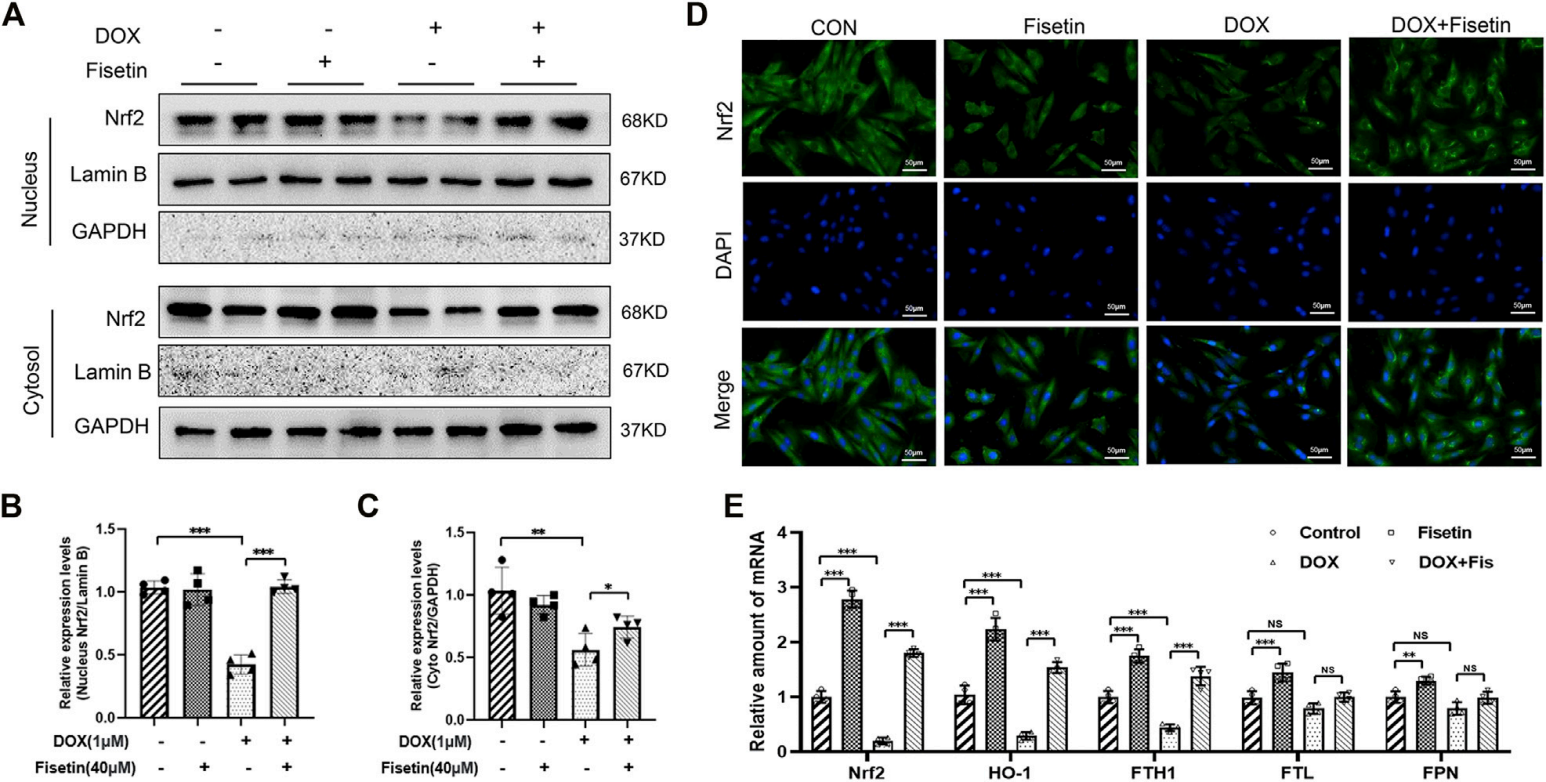
Fisetin activates Nrf2 and Nrf2 pathways in H9C2 cells. **(A)**. Western blot of nuclear and cytosol Nrf2 levels *in vitro*. **(B).** Nuclear Nrf2/labmin B ratio was quantified by ImageJ analysis. **(C)**. Quantification of cytosol Nrf2/GAPDH ratio. **(D)**. Detection of the Nrf2 expression and translocation using immunofluorescence (IF) staining in different groups. (Representative images, 200X, Scale bar = 50 μm). **(E)**. mRNA levels of Nrf2, HO-1, FTH1, FTL, and FPN were detected by real-time PCR in different group cells. The values are presented as mean ± SD. **p < 0.01; ***p < 0.001, NS, no significance; Nrf2, nuclear factor erythroid 2-related factor 2; Keap1, Kelch-like ECH-associated Protein 1; HO-1, heme oxygenase-1; FTH1, ferritin heavy chain 1; FTL, ferritin light chain; FPN, ferroportin; TfR1, transferrin receptor 1.

The transcription factor Nrf2, a master regulator of the cytoprotective effects against oxidative stress, plays a crucial role in ferroptosis regulation (Fan et al., 2017; Carpi-Santos and Calaza 2018). We subsequently determined whether the Nrf2 expression levels were changed in the DOX and DOX + fisetin groups. The data (Figure 4) indicated that the Nrf2 protein expression level was significantly decreased in the DOX group compared with that in the normal control group, whereas the DOX + fisetin group showed a significant increase in the Nrf2 protein level (Figures 4A,B). The results of immunohistochemical staining with the Nrf2 antibody confirmed these observations in heart tissues (Figure 4C). Moreover, the genes such as *HO-1, FTH1, FTL,* and *FPN* are thought to be the Nrf2 target genes involved in ferroptosis (Sun et al., 2016; Anandhan et al., 2020; Dong et al., 2020; Song and Long 2020). Our data indicated that the expression levels of *HO-1* and *FTH1* were significantly decreased in the DOX-treated heart tissues, whereas that of *FTL* was not dramatically changed, and *FPN* was observed to be markedly upregulated in the DOX-treated group. Interestingly, the expression of *TfR1*, which is not considered an Nrf2 target gene, was also decreased after DOX treatment (Figures 4A, B). Fisetin supplementation can increase the HO-1 and FTH1 protein levels in a dose-dependent manner. We observed similar results in the H9c2 cells and heart tissues; DOX decreased but fisetin increased the Nrf2 expression in both nuclear and cytosol and further enhanced Nrf2 nuclear translocation (Figures 5A–D) in the DOX-treated and fisetin supplementation groups. Real-time PCR indicated that fisetin could increase not only the expression levels of *Nrf2, HO-1*, and *FTH1* but also the *FTL* and *FPN* mRNA expression levels (Figure 5E).

### Fisetin Regulated Nrf2 by Upregulating the SIRT1 Protein Expression in the DOX-Treated Rats and H9c2 Cells

**FIGURE 6:**
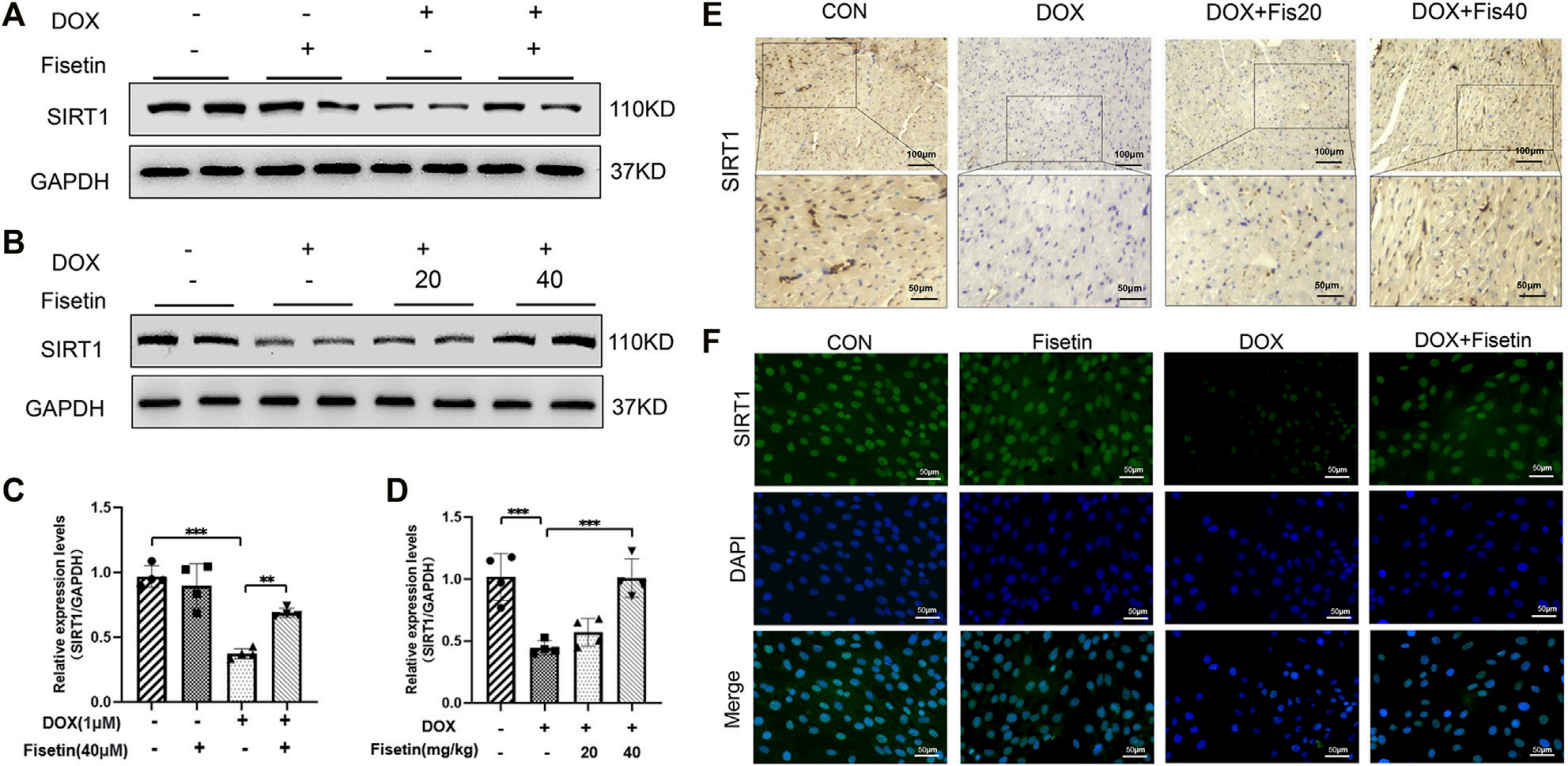
Fisetin attenuates ferroptosis *via* upregulating the SIRT1 protein expression in DOX-treated rats and H9c2 cells. **(A)**. SIRT1 protein expression levels in rats, as measured by Western blot. **(B)**. Western blot results of SIRT1 levels *in vitro*. **(C-D).** Quantification of **(A and B)**. **(E)**. Representative images of anti-SIRT1 staining by IHC in different groups (200X and 400X, Scale bar = 100 and 50 μm, *n* = 6 per group). **(F)**. Representative images of anti-SIRT1 IF staining (200X, Scale bar = 50 μm). The values are presented as mean ± SD. **p < 0.01; ***p < 0.001; SIRT1, Sirtuin 1.

To investigate whether the inhibitory effect of fisetin against ferroptosis is mediated *via* SIRT1, we measured the SIRT1 protein levels through Western blotting in both DOX- and DOX + fisetin-treated heart tissues and H9c2 cells. As shown in Figure 6, DOX downregulated the SIRT1 protein expression level, whereas the SIRT1 level was reversed by co-administration of fisetin (Figures 6A–D). Similar results were observed in IF staining by using the SIRT1 antibody (Figure 6F). Then, we performed IHC of the rat cardiac tissue sections to determine whether these alterations occurred in the DOX-treated heart tissues. The results also showed that the number of SIRT1-positive cells was remarkably decreased in the DOX-treated rats compared with that in the control rats (Figure 6E). As expected, co-treatment with DOX + fisetin prevented the heart tissue from DOX-induced effects of SIRT1 downregulation. Thus, SIRT1 was also a key factor for the protective effect of fisetin against ferroptosis *in vivo* and *in vitro*.

### Fisetin Alleviated DOX-Induced Ferroptosis *via* the SIRT1/Nrf2 Signaling Pathway in an SIRT1-Dependent Manner

**FIGURE 7:**
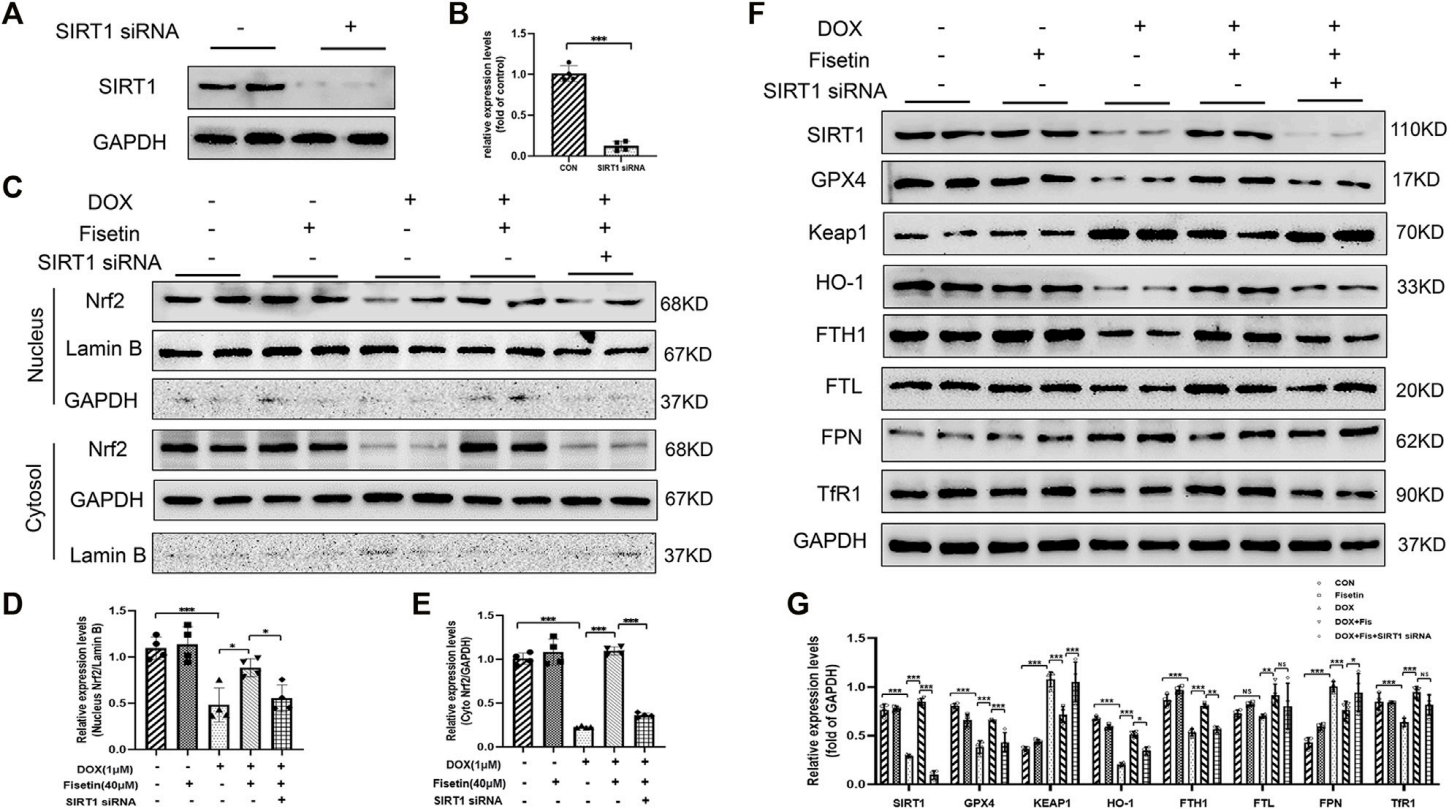
Fisetin alleviates DOX-induced ferroptosis *via* the SIRT1/Nrf2-regulated signaling pathway. **(A and B)**. Western blot results of SIRT1 protein levels after SIRT1 siRNA transfection. **(C)**. Western blot of nuclear and cytosol Nrf2 levels *in vitro* after treatment with DOX and/or fisetin, SIRT1 siRNA. **(D–E)** nucleus or cytosol Nrf2 protein from Western blot results. **(F)**. Protein expression levels of SIRT1, GPX4, Keap1, HO-1, FTH1, FTL, FPN, TfR1, and nuclear Nrf2 in H9c2 cells administrated with DOX and/or fisetin, SIRT1 siRNA. **(G)**. Quantification of SIRT1, GPX4, Keap1, HO-1, FTH1, FTL, FPN, and TfR1 protein from Western blot results (*n* = 4). The values are presented as mean ± SD. *p < 0.05; **p < 0.01; ***p < 0.001; NS, no significance.

Finally, we investigated whether SIRT1 acted as an upstream regulator of Nrf2 in mediating ferroptosis inhibition by fisetin. The H9c2 cells were transfected with SIRT1 siRNA to silence the SIRT1 expression (Figures 7A,B). Fisetin restored the DOX-induced low Nrf2 level in normal H9c2 cells; however, this effect was abolished under SIRT1 knockdown (Figures 7C–E). Consistent with the *in vivo* results, we noted that fisetin could reverse the effects such as low protein levels of HO-1 and FTH1 and a high protein level of FPN in the DOX + fisetin-treated cells compared with those in the DOX-treated cells. We also found FTL was slightly decreased (Figures 7F,G). However, these effects could be reversed by SIRT1 knockdown. The knockdown of SIRT1 also abolished fisetin-enhanced antioxidant gene upregulation (Figures 7F,G). Moreover, the upregulation effects of fisetin on GPX4 were inhibited by SIRT1 knockdown. We also found a significant increase in the Keap1 expression, which is the predominant repressor protein of Nrf2 critical for Nrf2 degradation, in the DOX-treated H9c2 cells (Itoh et al., 2010; Sihvola and Levonen 2017). Moreover, the MDA, GSH, ROS, and iron levels were detected in the SIRT1-knockdown cells and normal H9c2 cells treated with DOX + fisetin. The results indicated that fisetin could not inhibit ferroptosis during SIRT1 knockdown (Supplementary Figures S2A–D). Some studies reported that fisetin could increase the Nrf2 expression but did not mention SIRT1(Sandireddy et al., 2016; Wu et al., 2017; Zhang L et al., 2018). To determine whether fisetin directly interacted with Nrf2 or through the SIRT1-Nrf2 axis, we knocked down Nrf2 in H9c2 cells by using Nrf2 siRNA. We found that the Nrf2 protein level decreased (Supplementary Figure S3A); however, the SIRT1 expression level was not significantly decreased whereas GPX4, HO-1, and FTH1 expression levels decreased in the Nrf2-knockdown cells treated with DOX + fisetin (Supplementary Figures S3C–D). In conclusion, these results provided evidence that fisetin attenuated DOX-induced ferroptosis in the cardiomyocytes *via* the SIRT1/Nrf2 signaling pathway.

## Discussion

Doxorubicin (DOX) is a chemotherapeutic agent that is commonly used for the treatment of various types of cancer. However, the clinical application of DOX is limited due to its cardiotoxicity, which may lead to progressive, chronic, and life-threatening cardiomyopathy (Ewer and Ewer 2015; Nebigil and Désaubry 2018). Moreover, the effective treatments to improve cardiac dysfunction induced by DOX are lacking. Hence, finding an effective treatment remains an urgent requirement.

Flavonoids have emerged as powerful antioxidants. Fisetin, one of the flavonoids, is present in fruits and vegetables and has been reported to exert different beneficial biological effects such as antioxidant, anti-apoptosis, anti-inflammatory, antidiabetic, and neuroprotective effects (Zheng et al., 2008; Khan et al., 2013; Currais et al., 2014; Sundarraj et al., 2018; Zhang et al., 2019). Several studies have validated the cardioprotective effects of fisetin (Dong et al., 2018; Althunibat et al., 2019; Rodius et al., 2020). Ma et al. found that fisetin exerted significant cardioprotective effects against DOX-induced toxicity by inhibiting multiple pathways including oxidative stress (SOD, GSH, MDA, and NO), inflammation (COX-II, TNF-α, and IL-1β), and apoptosis (caspase-3) (Althunibat et al., 2019). In the present study, DOX treatment caused myocardial injury in the rats, which led to a decrease in the LVEF and LVFS and an increase in the LVIDd and LVIDs. However, these markers of myocardial function were attenuated by fisetin administration. Moreover, several studies have reported that ferroptosis is associated with DOX-induced cardiac toxicity, which involves multifactorial mechanisms (Fang et al., 2019; Tadokoro et al., 2020; Liu et al., 2021). Ferroptosis is dependent upon the intracellular iron concentration, accumulation of lipid ROS, and the lack of function of the lipid repair enzyme GPX4 (Wang Y et al., 2021). To assess DOX-induced ferroptosis, we found that DOX decreased the GPX4 expression and GSH level and increased the ROS and MDA levels *in vivo* and *in vitro*. The treatment with fisetin reversed these changes, indicating that DOX-induced cardiomyocyte injury was associated with ferroptosis and that this damage could be repaired by fisetin administration. Thus, we sought to investigate the pathway through which fisetin influenced ferroptosis *in vivo* and *in vitro*. The data of the present study demonstrated the pivotal role of SIRT1 in ameliorating oxidative stress and ferroptosis.

SIRT1, an NAD^+^-dependent class III histone deacetylase, is deemed to be responsible for the beneficial effects in the development and treatment of cardiovascular diseases (D'Onofrio et al., 2018; Kane and Sinclair 2018). The cardiac-specific overexpression of SIRT1 protects the heart from ischemia/reperfusion injury by negatively regulating pro-apoptotic molecules such as caspase-3, an endoplasmic reticulum (ER) stress downstream activator (Hsu et al., 2010). Studies have shown that the SIRT1 overexpression can inhibit ferroptosis-induced cardiomyocyte death in myocardial ischemia reperfusion (MI/R) (Hu et al., 2020). Moreover, SIRT1 has been shown to increase the cell resistance in DOX-induced cardiotoxicity (Yuan et al., 2018; Sun et al., 2020). SIRT1 can regulate and increase the Nrf2 activity (Fan et al., 2017). Nrf2 is a transcriptional factor that can regulate the antioxidant response elements (AREs) to coordinate the antioxidant system (Kaspar et al., 2009; Ma 2013). Under normal conditions, Nrf2 is constantly synthesized but maintained at low levels as it is competitively bound by Keap1 and subsequently deactivated by proteasome degradation through ubiquitination (Lu et al., 2017). The Nrf2-dependent cellular defense mechanism is activated after stimulation, leading to the separation of Nrf2 from Keap1 and facilitating entry of Nrf2 to the nucleus to activate its downstream genes (Tuo et al., 2018). *HO-1, FTH1, FTL,* and *FPN* are the most important Nrf2 target genes involved in ferroptosis (Anandhan et al., 2020; Dong et al., 2020; Song and Long 2020). *HO-1* can reduce intracellular ROS production (Kobayashi and Yamamoto 2005). A study reported that DOX-induced cardiotoxicity is mediated through the Nrf2/HO-1 axis (Fang et al., 2019). Although FTH1 and FTL have the ferroxidase activity and can convert Fe^2+^ to Fe^3+^, which is important for iron entry into the ferritin and iron storage, FPN is the only known transmembrane exporter of nonheme iron that transports Fe^2+^ out of the cells (Chen et al., 2020). A high intercellular iron concentration leads to lipid peroxidation and eventually ferroptosis (Chen et al., 2020).

Fisetin has been showed to increase the SIRT1 expression. Kim. S et al. found that fisetin facilitated SIRT1-mediated deacetylation of peroxisome proliferator–activated receptor (PPARγ), enhanced the association between SIRT1 and the PPARγ promoter in 3T3-L1 cells, and therefore increased the SIRT1 level (Kim et al., 2015). Liou et al. showed that fisetin increased AMPKα phosphorylation, which was further associated with a high SIRT1 expression (Liou et al., 2018). Singh et al. found that fisetin upregulated the expression of autophagy genes (Atg-3 and Beclin-1) and SIRT1 in rats (Singh et al., 2018). Kim. A and Kim. S et al. also found that fisetin modulated the transcription factors of the FOXO family members such as FoxO1 and FoxO3a to activate the SIRT1 expression in 3T3-L1 and human monocytic cells (THP-1) (Kim et al., 2015; Kim et al., 2017). Fisetin exerts some beneficial anti-inflammatory, antioxidant effects, and cardiovascular protective effects *via* SIRT1 activation.

In our study, SIRT1 and Nrf2 expressions were downregulated in the DOX-treated rats and H9c2 cells, and the present study results showed that fisetin significantly increased the SIRT1 expression and Nrf2 activity *in vivo* and *in vitro*. Fisetin restored SIRT1 downregulation and alleviated ferroptosis by increasing the GPX4 and GSH levels. Under DOX stimulation, the Nrf2, *HO-1*, and *FTH1* expression levels were decreased significantly, but the Keap1 protein expression level was increased. The effect of fisetin on the Nrf2 upregulation and nuclear translocation led to an increase in the expressions of *HO-1* and *FTH1* genes at both mRNA and protein levels in the DOX-induced rats and H9c2 cells. SIRT1 knockdown downregulated the expressions of Nrf2, *HO-1*, and *FTH1*. Thus, our data demonstrated that fisetin attenuated DOX-induced cardiomyopathy associated with ferroptosis through the SIRT1/Nrf2/HO-1 pathway and the FTH1 axis.

Although the present study revealed the fisetin’s protective effect against DOX-induced myocardial ferroptotic cell death *in vivo* and *in vitro*, the study still has some limitations. DOX downregulated the SIRT1/Nrf2 signaling pathway, whereas fisetin could restore this DOX-induced effect in the cardiomyocytes, and the Nrf2 target genes were activated after fisetin supplementation. Our data indicated that the *Nrf2*, *HO-1*, and *FTH1* expressions were significantly increased, but the *FTL* expression exhibited no remarkable change. Surprisingly, the *FPN* expression increased after DOX treatment and decreased after fisetin administration. Another gene *TfR1* that functions in iron uptake is not an Nrf2 target gene but exhibited a similar expression pattern as *HO-1*. These data indicated that some other pathways and mechanisms may be involved in the protective effect mediated by fisetin. Therefore, further studies are warranted to evaluate these effects in detail.

## Conclusion

The present study demonstrated that fisetin, as an anti-ferroptosis compound, can attenuate doxorubicin-induced cardiomyopathy *in vivo* and *in vitro*. Fisetin improved the myocardial function by alleviating cardiac dysfunction, ameliorating myocardial fibrosis, mitigating cardiac hypertrophy in DOX-induced rats, and increasing the expressions of SIRT1/Nrf2 pathway genes, *HO-1* and *FTH1*, in rats and H9c2 cells. Our findings suggest that the SIRT1/Nrf2 signaling pathway is the main pathway involved in attenuation of doxorubicin-induced cardiotoxicity by fisetin, which can be used for ameliorating chronic cardiomyopathy.

## Data Availability

The original contributions presented in the study are included in the article/Supplementary Material; further inquiries can be directed to the corresponding authors.

## Supplementary Material

Supplementary Figure S1
**(A)** Western blot results of GPX4, nucleus Nrf2, and cytosol Nrf2 protein levels in H9c2 cells administered with DOX or Fer-1(*n* = 4). **(B–D)** Quantification of **(A)**. The values are presented as mean ± SD. *** *p* < 0.001;

Supplementary Figure S2
**(A-D)**. MDA, GSH, ROS, and iron concentrations were detected in H9c2 cells treated with DOX and/or fisetin, SIRT1 siRNA, respectively(*n* = 5). The values are presented as mean ± SD. *** *p* < 0.001; NS, no significance.

Supplementary Figure S3
**(A-B)**. Western blot results of Nrf2 protein levels after Nrf2 siRNA transfection. **(C)**. Western blot results of Nrf2, GPX4, SIRT1, HO-1, and FTH1 protein in H9c2 cells administrated with DOX and/or fisetin, Nrf2 siRNA. **(D)**. Quantification of Nrf2, GPX4, SIRT1, HO-1, and FTH1 protein analyzed by Image J (*n* = 4). * *p* < 0.05; ** *p* < 0.01; *** *p* < 0.001; NS, no significance.
