# Supplementary figures and images for: Fisetin Attenuates Doxorubicin-Induced Cardiomyopathy In Vivo and In Vitro by Inhibiting Ferroptosis Through SIRT1/Nrf2 Signaling Pathway Activation
Source: Front Pharmacol. 2022 Feb 22;12:808480. doi: 10.3389/fphar.2021.808480 (PMC8902236; doi:10.3389/fphar.2021.808480)

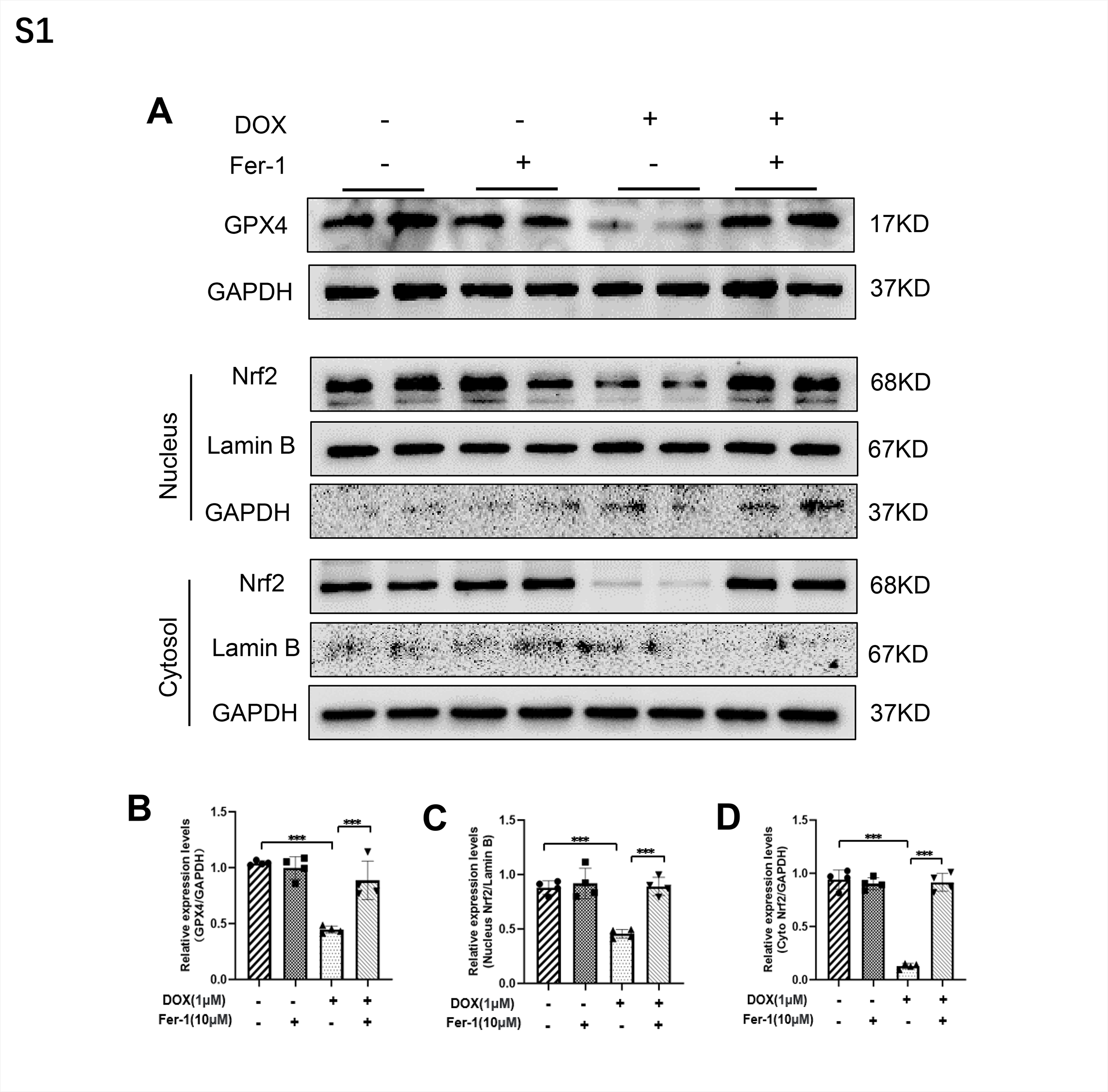

Supplement: Supplementary file 1 [file DataSheet1.zip › Figure S1.TIF]

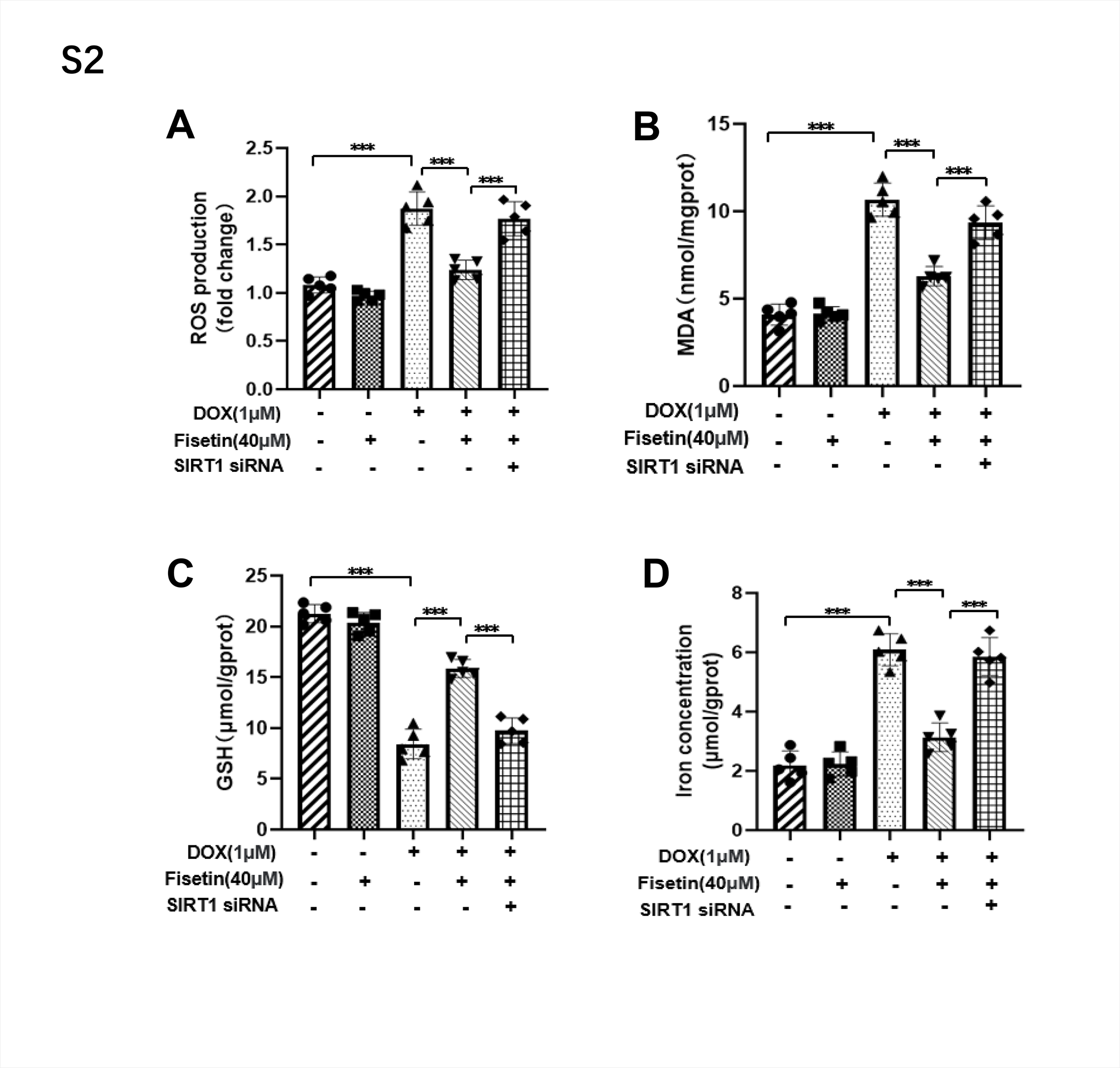

Supplement: Supplementary file 1 [file DataSheet1.zip › Figure S2.TIF]

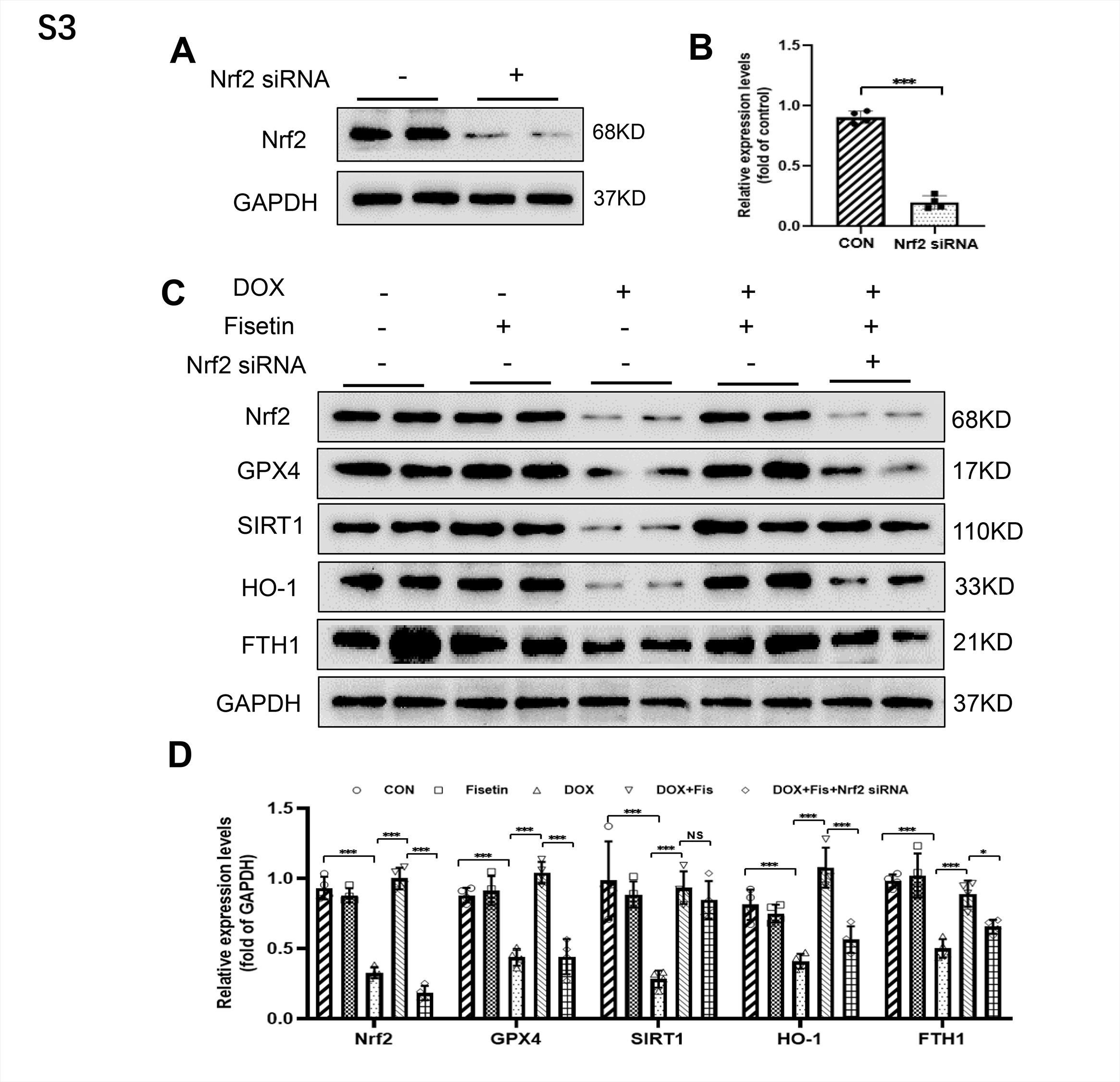

Supplement: Supplementary file 1 [file DataSheet1.zip › Figure S3.TIF]
